# Interpretive Methods in Disability Studies: Dyslexia Inflected Inquiry

**DOI:** 10.1177/10778004241254394

**Published:** 2024-06-03

**Authors:** Tanya Titchkosky

**Affiliations:** 1 University of Toronto, Ontario, Canada

**Keywords:** interpretive methods, impairment experience, dyslexia, disability-perception, disruption, common-sense, normative flow

## Abstract

This article explores how disability studies can take shape as an interpretive method and how disability-perception can influence this. My exploration is organized in relation to the following question: In what ways might attention to dyslexia as an interpretive act inflect social inquiry? I treat interpretive methods as a form of inquiry that attends to the social activity of interpretation itself and I regard dyslexia as part of such activity. A key issue for such inquiry is how to methodically engage appearances *as* an interpretive encounter: That is, how can we make the taken-for-granted activity of *perception as interpretation* available for reflection and keep the subject ∞ object chiasma* relation a primary focus? Disability studies is a starting point for such inquiry in at least two ways: (a) it brings to attention the ways in which people interpret disability and (b) it considers how impairment experience itself is an interpretive modality that can momentarily disrupt the normative flow of common-sense, revealing aspects of the act of interpretation, and making it available for reflection. This article will show how the perception of disability as well as disability-perception can be regarded as enacting a “pause” in the everyday flow of common-sense and, thereby, encounter interpretive acts as an occasion for further inquiry. I turn to descriptions of perceptions and experiences of dyslexia as interpretive scenes where the normative order of ordinary interpretation can be revealed. I address various ways that dyslexia is described as a disruption to the normative order of language, especially the written word, print-language, even as the term dyslexia is used as a sense-making-device to reassert the primacy of normative expectations and values in literate-culture. As a sense-making-device imposed from without or as experience that seems to come from within, or as both, the appearance of “dyslexia” serves as a primal scene for uncovering the ways in which the social order of interpretation works.

*Chiasma is not a dyslexic rendering of “chasm.” Instead, *chiasma* refers to the crosswise relation between concepts and structures that rely on each other for their meaning, for example, reading and readers; subject and object.

## Introduction

This article explores dyslexic inflected inquiry as an interpretive method and how such a method is shaped by disability-perception. I will turn to historical and professional depictions of dyslexia understood as objective as well as more experiential descriptions understood as subjective, to conduct this exploration of dyslexia inflected inquiry. Insofar as dyslexia can be experienced as a disruption to the normative order (even as it is used to make sense of departures from expected norms of literate culture and its print-languages), it serves here as a social scene of exploration where we can uncover some of the features of interpretation itself. I treat dyslexia-depictions as social scenes where the normative order of interpretation can be revealed through a disability studies orientation. Disability studies holds that limit and lack do not represent the whole meaning of disability and claims that disability is something more than a problem in need of a solution. Disability is, instead, something from which we can learn about the ordinary composition of daily life.

Interpretive methods can be characterized as a way to focus on the social activity of interpretation. There are a variety of critical studies, such as Indigenous, Black, feminist, queer and trans studies, as well as disability studies that make use of interpretive methods in their inquiries. In contrast to qualitative and more positivistic inquiry that proceed from a defined sense of identity or meaning, interpretive methods places it critical focus on how meanings are made such that categories of people, places, things appear as they do (Yanow, 2003; Yanow & Schwartz-Shea, 2014). Research that seeks causes for various categorizations of people, but does not engage aspects of identity or meaning expressed by these categories as forms of interpretation, leaves the social character of perception (interpretation) unexplored. A key feature of interpretive methods is its orientation toward de-naturalizing the social categories through which the meaning of persons is forged (Ahmed, 2006; Gilroy, 2002, 2005; Wynter, 2006). This methodological orientation begins with the premise that interpretive acts are taken for granted aspects of the meaningful appearance of all social life. Social life includes categories of people, people as problems, facts about these people, and other objective claims, as well as life understood as subjective, such as experience, thoughts, feelings and the like. Through the recognition of the essential relation between subject and object (Merleau-Ponty, 1945/1962, p. xxii), interpretive methods are committed to attending to whatever appears as human-made as a scene of interpretive action. This scene thus becomes the primal focus of inquiry (Healey & Titchkosky, 2022; Michalko 1998).

It is crucial to consider how to methodically engage appearances *as* an interpretive encounter, and to ask: how can we make the taken for granted activity of interpretation available for reflection as a scene of action? My paper will show how the perception of disability as well as disability-perception can be understood as an enactment of hesitation in the regular flow of interpretation. This hesitation permits us to engage the taken for granted activity of interpretation (as theorized by Al-Saji 2018; Asenjo, 1988). Instead of ignoring the relation between the subject ∞ object as a form of knowledge production, interpretive methods attends to their essential inter-relatedness. Hesitation, a form of the epoché, supports attending to the perception of disability in everyday life (Schutz, 1973). Interestingly, it is such *hesitation* that is often read as indicative of disability. Insofar as hesitation is revelatory of disability-as-perception, it reveals, too, the taken-for-granted aspects of the interpretive processes that produced disability-designations in the first place.

In what follows, I turn, first, to descriptions of dyslexia as it became a fact of history within Western English-speaking literate cultures. I then address more current programmatic descriptions of dyslexia that are oriented to taking actions to reduce the effects of dyslexia on children. Finally, I explore the experience of confusion and difficulties that I call “my dyslexia” as well as other people’s narratives of what goes under the banner of subjective dyslexic-experience. I characterize these three interpretive scenes as historical, professional, and personal depictions of dyslexia. Engaging these scenes through an interpretive disability studies perspective allows us to not only recognize the subject ∞ object chiasma^1^ relation, but to focus our inquiry on this relation. Directing inquiry to such concerns serves as the basis for narrating some of the ways dyslexia-inflected inquiry can proceed. The perception of disability as well as disability-perception are hesitations in the everyday flow of interpretation making possible further inquiry into the meaning of human relations.

## Dyslexia—An Origin Story

My heart is heavy, my eyes are dim,

my feet have stumbled, my arm is weak,

my hand is slow,

I cannot write my tablet,

I cannot read my tablet,

I do not know what is in my tablet.

“The Lament of Nanni,” 2000 BCE (Alster, 1972)

Difficulty in reading is a concern as old as writing itself and records of this concern can be found in ancient texts (Manguel, 1996). However, difficulty reading understood as a specific individual impairment does not appear until the advent of mass-literacy (Campbell, 2013). Unlike the ancient text above, difficulty reading as an impairment, post-mass literacy, is not connected to aging, physical impairment, or poor environmental conditions. Dyslexia, once known as “word blindness,” is typically used by professionals today to reference some peoples’ difficulty with “accurate and fluent word reading,” de-coding, spelling, and other literacy difficulties (Kirby & Snowling, 2022, p. 4; Duke et al., 2004). The appearance of dyslexia is a form of interpretive action, historically situated within the wider global authority of print-language.

Originating as a way to address white middle and upper class boys regarded as “otherwise bright” but failing to read as well as do their peers, dyslexia served to hold open a spot for those deemed not impaired enough to fit the categories of disqualification operating at that time (such as feeblemindedness, imbecility, and idiocy), and yet also not fit-enough to assume their ready-made place in the cultural structures reliant on the language of print (Kirby & Snowling, 2022, pp. 19, 29). This thus indicates the ambiguous and special place of the dyslexic child. By the end of World War II, “dyslexia” is firmly established as an interpretive category available to make sense of, treat, and position the “otherwise bright” white boy of means who struggles with “his letters” (Kirby, 2020; Kirby & Snowling, 2022, pp. 28–29). “His letters” signifies ways of reading and writing sanctioned by the state as it implemented mass literacy.

Mass literacy occurred in Britain and some of its colonies through mandatory education enacted in the 1870s. Around the same time, mandatory education is enacted in European nations, their colonies, and many U.S. states. According to Benedict Anderson (1983, p. 44), “print-language” generally, and not *a* language in particular, has been key to the invention of nationalism and for laying the bases of a national consciousness, which necessarily includes conceptions of literacy itself. This sense of literacy as tied to subject formation, citizenry, and the maintenance of the nation state has now become so well established that it is almost beyond notice.

The sense that literacy harbors the productive power to forge the meaning of persons and places is expressed regularly in research and media reports of the following ilk: “Nearly half of adult Canadians struggle with literacy—and that’s bad for the economy” (CBC Radio, 2021). Such headlines express the taken for granted sense that national identity is tied to literacy measured within a population, and that all of this is tied to the well-being of the nation state. The introduction of literacy-rates by nation-states serve as a process through which populations are delineated and assessed while drawing out predictive-connections to economic, and other forms of national and personal well-being. Let us consider this social process further.

Mandatory mass education can be understood as “one of the most common and recognizable forms of state socialization,” a socialization occurring around the globe but not in equal measure (Jackson, 2022, p. 1).^2^ The introduction of mandatory mass education varies depending on a “complex recipe of ingredients” including notions of race, gender, religion as these play out through imperialism and colonization, and other economic and “civilizing” projects (Jackson, 2022, pp. 1, 2). The need to not only make sense of, but systemically manage those who cannot read or struggle to do so, is part and parcel of mandatory mass education. Ways of organizing conceptions of those learning to read and those who struggle to do so are comprised of a complex recipe of ingredients no less tied to nationalism, racism, eugenics, and other ideologies formative to mass education itself.

By the 1930s, mass education and national literacy endeavors in Europe and America are intertwined with the logics of eugenic and/or racialization processes. Literacy education and testing, for example, are used to not merely mark those of ability and those without, but also to sustain existing racist conceptions of who possesses the ability to learn and who does not. This conception of (individual[ized]) ability serves to socialize people to accept the normalcy, even necessity, of doing so for the sake of the well-being of the nation-state. “Adapted education,” for example, “most strongly associated with education in Africa” was developed to help establish imperial aims and interests while responding to humanitarian, religious, national, traditional and other competing interests often under the banner of national development (Jackson, 2022, pp. 6–7, see also Césaire, 1972; Dei & Adjei, 2024; Freire, 1983; Kawano, 2022; Slee, 2004). Dyslexia is folded into this mix as a category of interpretation that takes shape as a way for professionals to make sense of some of those who struggle to read (Gabel, 2005; Jenkins, 1998). The sense that dyslexia does not arise as an interpretive possibility until state or imperial powers mandate mass education provokes further consideration (see Goodley, 2020 for further discussion of the capitalist making of disability).

## The Complexity of Defining Dyslexia

Forms of adapted and special education emerge with the advent of mass education. Various categories of persons also emerge that fit these forms, categories such as the developmentally delayed, those with special needs, and those who experience difficulties while learning but who might otherwise be deemed “normal” participants within education. Dyslexia holds a “special” place within these categories of difference, differences that contemporary structures of education rely on, test for, and reproduce.

The special place of dyslexia in this disability matrix can be gleaned from definitions of it in circulation around the globe today. For example

An aspect of the special character of dyslexia lies in how it functions differently than do other disability labels. Such labels often excuse people from “normal role obligations.” Disability is typically structured as a sick-role, a medically legitimated “can’t do” unrelated to a lack of will (Parsons, 1951, pp. 434–439). Instead of a structured disqualification, dyslexia is structured more like a qualified inclusion. The label of dyslexia serves to suggest that a person is “overall intelligent, but …,” or a person is “able to use higher level language skills, but …,” or they “can comprehend, but …” This reflects a historical continuity with the “otherwise bright or intelligent” child through which “word blindness” was first associated and it also reflects the everyday sentiment, “They’re smart, but there is something not quite right.” Dyslexia engenders an *unexpected* problem with words and their sounds and symbols, but this unexpectedness is encountered only insofar as the person more or less appears as the learner that they are expected to be, which is a normal learner (Peters, 2005). Even in the “21st century definition of dyslexia,” which asserts that dyslexia is a genetically based difference in thought itself, their remains the “smart, but …” sentiment, for example, “Generally a dyslexic cognitive profile will be uneven when compared to a neurotypical cognitive profile.” (MadeByDyslexia, n.d.).

Regardless of its cause, dyslexia is typically regarded as unrelated to vision or comprehension, and is further regarded as something that can be addressed through various interventions that exceed the act of reading itself. “A good indication of the severity and persistence of dyslexic difficulties can be gained by examining how the individual responds or has responded to well-founded interventions” (British Dyslexia Association [BDA], 2010). This means, ironically, that if dyslexia-oriented interventions work to make someone a better reader, it is likely because the person is dyslexic, and if these interventions did not work, it is likely because the person is dyslexic. The ubiquity of the possibility of being labeled dyslexic is again related to its special status as a diagnostic category that frames those who struggle to read within a technical version of reading.^3^ This sense of reading is comprised of technical interventions, sometimes called the “science of reading,” whereby people are trained on some component parts of reading, such as phonemes and word recognition. The ubiquity of potentially problem readers is met with a rather singular sense of reading along with contained and constraining solutions.

While many definitions of dyslexia and/or discussions of its causes in circulation today suggest that it is not related to comprehension or vision, they intimate that, if left untreated, dyslexia can seriously affect how a person experiences, understands, and envisions both the world and their place in it. For example, an influential Ontario Human Rights Commission (OHRC, 2022) report, called *the Right to Read*, asserts that untreated dyslexia can lead to “dropping out of school and developing psychiatric problems” as well as to “increased risk of underemployment or unemployment, relying on social assistance, poverty, homelessness, criminalization and even suicide” as well as crime (pp. 9–11).^4^ These claims are in wide circulation across the province of Ontario including the Toronto District School Board, which is one of the largest school boards in North America. While untreated dyslexia is thought to lead to deviance from normal role obligation, it does not provide a blanket disqualification from these obligations in the same way as do other disability labels. The person labeled with dyslexia is positioned as both inside and outside the normative order of literate culture generating a sense of ambiguity. This is what makes dyslexia hold a “special place” in the contemporary use of disability categories of difference, such as developmental delay, visual and/or hearing impairment, physical impairment, and mental illness.

The special place of dyslexia in the matrix of disability categories is not only due to its ambiguous structure; it is also due to its differential use. Some nation states, for example, adopted and promoted dyslexia as a way to make sense of difficulty reading and others have not (Kawano, 2022, p. 108). The use of the label dyslexia varies within regions, provinces and territories of nation states. For instance, starting in September 2023, the province of Ontario, Canada subjects all students to a “… common, standardized evidence-based screening assessments twice a year from Kindergarten to Grade 2, to identify students at risk for reading difficulties for immediate, early, tiered interventions” (OHRC, 2022, p. 12). This mass screening program is reliant upon a singular sense of what reading is, namely, de-coding based on phonic recognition (Cummins, 2022). This is not the same in other provinces or territories where, for example, there is a focus on developing literacy programs in over a dozen languages, many of which are not phonic-based (Government of the Northwest Territories, n.d., pp. 8–9). This suggests that in some locales, reading programs, more so than individual children’s capacities (and incapacities), are under state scrutiny and assessment. Examining literacy programs in relation to dyslexia is a far different strategy than pursuing MRI scans, genetic markers, or other bio-technologies where there is an intense technological focus on the individual regardless of the consistency of results. Insofar as literacy and screening programs of any sort can be regarded as part of state socialization, the delineation of reading difficulties serves to instruct their adult populations to “read” for reading troubles by, for example, marking a difference between individual incapacity, programmatic differences, or structured inequalities (Titchkosky, 2008, p. 345). Added to this mix are debates as to whether dyslexia is a “disability” or just a learning difference fully amenable to remediation (Barden, 2011).

Within all these debates, there are assumptions about what learning to read looks like, how learning works, as well as what should matter about reading difficulties. The subsequent use of the label of dyslexia, as well as variations in its use, shows the socially constructed character of dyslexia, its relation to norms of learning, individualized conceptions of intelligence, as well as the sheer complexity of the movement and meaning of interpretation itself in making the meaning of people and of problems. Still, there is much to learn about what this label is doing for contemporary times as well as what the special position of the dyslexic figure might have to recommend to the pursuit of interpretive methods of social inquiry within literate times.

## Dyslexia as an Interpretive Device

The category “dyslexia” is an interpretive device that arises in relation to the interpretive act of reading and it becomes a possible identity category only upon the advent of the cultural complex of mass literacy. Its utility as an interpretive device is, moreover, highly contingent on a variety of social and political factors. While dyslexia is a category that is used to problematize the reader understood as failing to read in a normal fashion, it leaves unproblematized reading conceived in technical terms as “word recognition and decoding.” Reading conceptualized in this way does not acknowledge the power of the mass literacy cultural complex to socialize people to perceive reading through dichotomies, one between reader and reading as well as one between problem readers and those who read without a problem (Davies, 1998; Heap, 1991; Jenkins, 1998; Meek, 1992, Titchkosky, 2008). In this way, the use of the interpretive device of dyslexia downplays complex or contradictory meanings of reading while problem-readers are crystallized into fact.

Dyslexia inscribes trouble onto individuals, prescribing solutions, thereby lending certainty to the idea that some people should be evaluated for their reading problems while the norms of literacy are understood as almost beyond consideration. It is, after all, simply good to read, yet, as to whether there is a good conception of reading at play, is typically unquestioned. There are, for example, debates as to the best way to teach reading, but there are almost no debates as to the status of reading’s place as a necessity, that is, as a taken-for-granted basis for participation in daily life. (Is it possible to imagine reading otherwise?) A contemporary consequence that both supports and follows from a taken-for-granted relation to literacy as a foundational and self-evident good, is the creation of a clear dichotomy between an objectively given “science of reading” and people who have problems reading.

The culture of literacy has, in other words, established a working dichotomy between reading and the problem of being a reader as well as between good-readers and struggling-readers and socializes people to understand reading through such dichotomies. Approaching reading with an interpretive methods frame, however, allows us to “hesitate” in the face of proclaimed self-evident dichotomies as a way to elucidate relations to reading within a culture that assumes literacy as one of its foundations (Al-Saji, 2018). A focus on interpretation permits us to understand reading as a relation between reading and readers rather than as a dichotomy. Instead of treating dichotomy as a given or as something to be dismissed as an unnecessary contemporary myth, interpretive methods suggests treating readers and reading as in need of further engagement and, in this way, perhaps perceive the subject ∞ object chiasma. This chiasma forms a perceptual field with nodes of attention (problem readers), dis-attention (culture of literacy), as well as what typically remains invisible, namely, the organizing force of the chiasma itself (an intimate relation since there can, literally, be no reading without readers).

To perceive this chiasma is to witness what may at first appear as a subject object divide *as the unity it is*. It is to momentarily perceive a flash of a “unity” between the “extreme subjectivism” of individuals who struggle to read and the “extreme objectivism” of literate cultures that conceive of reading in technical terms (Merleau-Ponty, 1945/1962, p. xxii; see also Asenjo, 1988). When readers and reading are perceived in relation to one another, it becomes possible to recognize how literacy functions as a vast and nuanced mediation of cultural values and processes that people take-for-granted. The power generated through a taken-for-granted relation to the culture of literacy is such that the identity “Reader” may only come to consciousness with the advent of an accident, disease, aging, or, more recently, dyslexia. Perceiving the chiasma of literacy makes visible some of the threads of the closely woven “world” of literacy in contemporary times (Merleau-Ponty, 1945\1962, p. xxii). Further, perceiving the intertwining of subject and object is tied to the interpretive methodological commitment to conduct a phenomenology of the concept,^5^ in this case a phenomenology of the term “dyslexia.”

Attending to the concept means coming to perceive the interpretive possibilities in the development and use of the category of dyslexia. Proceeding with an interpretive methods frame means perceiving the socio-historical scene of dyslexia as comprised of relations between persons who struggles to read and the mass literacy of literate culture. This scene includes a functional normative dichotomy in the middle of an ageless and seemingly magical phenomenon, namely, reading. Some readers are positioned to discern problem-readers; moreover, these problem-readers represent limit and are fitted into a professional diagnostic mill that represents help and potential. This mill not only assesses people for their reading problems, it also manages wider conceptions of reading itself, conceptions such as the “science of reading.”

As a way to further an interpretive analysis of this mill of professionalism of reading for reading problems, I turn now to a personal experience of troubled reading.

## Dyslexia—My Problem

Perhaps the most cogent consequence of the professionalization of literacy is its focus on reading as an individual phenomenon, instead of a social one. I begin with a depiction, a personal one, of dyslexia, as a way to demonstrate that professional approaches to reading overwrite the way that something like a dyslexic-experience is social through and through. Engaging dyslexic experience as social nurtures the possibility that this experience of reading troubles offers much not only to interpretive methods of inquiry but to the question of interpretation itself. This offer is, however, contingent upon treating dyslexic experience, including the remedial action that follows it, as something to reflect on rather than as merely something to do.

**I Read**

The word is OHIO; maybe IOWA

The word is right there on the page—

To be read, to be read aloud …

I read. My mouth fills with marbles; marbles and marbles of vowels swirling round.

Sounds rolling through my mouth, spilling out

Hi oh ho

Hi ho ho

Wa I O

WA hi ho

Oh no.

Look away, look away. Shake your head. Turn away from the vowels.

Look to the sky …

and there,

I grab a memory of how to say this word of many vowels.

Iowa—clear as a bell!

Ding dong,

the witch is dead,

the word of vowels is finally said.

Iowa.

And then … I laugh at my difficulty of saying what is, after all, only a four-letter word

Ohio.

I struggle to read and especially to read aloud. And, the struggle remains even after writing about it and reading it aloud. These two particular words, OHIO and IOWA, are such a jumble that the thousands of miles between these American place names visit me as if one singular trouble-some locale wherein I experience “I can’t” and “What’s wrong with me?!” Moreover, there is Idaho, as well as ignominy, imagery, impatience, and countless other words, that may or may not begin with vowels, all of which bring me to the same locale – a struggle with discerning sound and sense.

I can and do read and write about my struggle with reading and writing, and my engagement with this struggle gives me something more than difficulty. After all, I creatively engage words and attempt to get closer to how they have been made to mean, or I swap one word out for another, read with dramatic flare, or tune into the greater ecosystem of word-use that comes along with any communicative moment; or, I do all of this. Nonetheless, the potential to mistake one word for the other, to confuse them in the struggle to read them, remains an always present possibility. Is this not, however, true of all reading by any reader?

We can note that *how* words, vowels, phonemes sound, and how their meaning are jumbled might be unique to people like me, to people who are understood as experiencing reading difficulties. The act of reading is, however, always tied to the potentiality of struggle, mistake, and confusion. There is no reading experience without such risks. The struggle to read, along with mistake and confusion, is an integral part of reading, which proficient readers also experience but may not need to attend to. Still, the struggle to read is to come in touch with *how* the interpretive act is accomplished through innumerable social expectations. How I read includes a sense of difficulty not only because I get it wrong (is it Iowa or Ohio and how to read either word correctly?) but also because the existential anxiety of the complexity of communication itself shimmers and shakes in my struggle. It is in this shimmering and shaking that I, we, can come in touch with the interpretive act of reading. This is not a science of reading, but it is a struggle. This shimmering and shaking leads to an existential anxiety: “Can I read? Can I understand? Can I read aloud so that you can understand?” which means—can I be in communication with that which is not me – with the text, the author, the scene?

The act of reading, as depicted above, points toward something more and other than an individual’s “decoding skill-set” meeting the objective text, comprised of phonemes, resulting in words read aloud. Still, words are read and read aloud. This involves some relation to a kind of flash-card recognition of the place names (OHIO/IOWA) as well as phonemes from which they are comprised (OH/IO/WA). Sounding out phonemes, however, like finding the flashes of word recognition cannot be all that reading is. Containing reading within a technical orientation leaves out, or more to the point, overwrites and diminishes the experience of reading which includes struggle as well as mistakes and confusion since reading is an interpretive act. *If* struggle, mistake, and confusion are removed from reading what remains would be a dis-utopian fantasy of a place of certainty without interpretation—a place we might call the country of professionalism. Professionalism assumes that communication can be “consistent, coherent, and clear” and these 3Cs are proofed through reading.

## The Country of Professionalism

The country of professionalism organizes the struggle to read in a hyper-individualized manner where reading appears as a strictly individual problem. This despite the social fact that reading is an interpretive act always already within a community of readers. I turn now to a consideration of how a commitment to interpretive methods and its preservation of the subject ∞ object chaismatic relation between readers and reading need not be derailed by the hyper-individualization of reading.

In *My Dyslexia*, Philip Schultz (2011, p. 26) puts his individual struggle to read in this way:

The act of translating what for me are the mysterious symbols of communication into actual comprehension has always been a hardship to me. I often read a sentence two or three times before I truly understand it; must restructure its syntax and sound out its syllables before I can begin to absorb its meaning and move on to the next sentence. And when I make the mistake of becoming aware that I am reading, and behaving in a way that enables this mysterious, electrically charged process to take place, my mind balks and goes blank and I become anxious and stop.

Making the “mistake” of noticing that he is reading, which means engaging in that intimate chiasma relation of self/word/world, Schultz (2011, pp. 20, 37) turns to his mind, or “the brain” to account for this mistake. He says,

I was suffering, to a noticeable degree, by the very manner in which my mind received and then attempted to process information—I was becoming conscious of the convoluted way my mind worked ... (20). My brain wouldn’t obey me, nor my parents or my teachers. If I had trouble learning to read a clock, know my left from my right, hearing instructions—things everyone else seem to do easily—how could I trust my own thoughts or anything about myself. (37)

The mystery of symbolic communication—an ageless mystery—can appear as a matter of clear coherent comprehension when perceived through professionalized reading concerns. This quest for comprehension seizes on meaning understood as objectively there waiting to be readily grasped by those able to do so. Regarding reading as the “act of translation,” as a “restructuring,” moves a reader away from the mystery of symbols of communication and toward comprehension which means absorbing enough sense so that the reader can “move on to the next sentence.” This capacity is key to normal participation in the movement of daily life of literate culture. Still, there is more to reading than accurate comprehension necessary for moving on to the next sentence, and more than the invocation of the “brain” allows for.

The task of reading understood as translating, restructuring, and moving along can be confounded by the recognition of the mysterious “electrically charged process” of reading itself. Thus, reading must be more than translation of symbols, mysterious or not, restructured into meanings that moves a person from one sentence to the next. But Schultz says that he stops reading as soon as he engages reading as the mysterious symbolic communication that it is. Even though Schultz balks at this electrical charge, what can be understood as the feel of interpretation, reading remains more than an act of translation on the way to correct comprehension and the next sentence. *Reading is the mystery of coming in touch with someone we are not and in touch with somewhere we are not and with things of which we are not certain*. Even though Schultz experiences reading as the “convoluted way” his mind works, indicative of a brain that “wouldn’t obey,” *this* hyper-individualized version of reading still puts him in touch with that of which he is not certain, namely, himself. “[H]ow could I trust my own thoughts or anything about myself?”

It is, however, only when we stop engaging reading as merely a mechanical translation of its component parts and actually feel our difference from the text that we become immersed in the mystery of reading. The meaning on the page is not, for example, *in* the phonemes, words or sentences or any other technical part of reading however necessary those parts may be. The symbolic communication that is reading means the risk of struggle, mistake, and confusion are always present since to read is to be in touch with what one is not and this always includes the possibility of balking, blanking, becoming anxious and stopping. The professionalization of reading transposes the symbolic communication that reading *is* into accurate and inaccurate decoding and correct and incorrect comprehension. Instead of mistakes existing between readers and what is read, and rather than being in the midst of a community of readers who embody confusion in the act of communication, the professionalization of reading posits the sense that there is simply something objectively there that needs to be comprehended and there are subjects who do this and others who do not.

And, at some level, I know there is nothing not true about this.

For comprehension to be the singular truth of reading requires that the reader dis-attend to the mystery of reading and become alienated from the wonder that there is a collective need to communicate which can be actualized through reading. Such a sense of mystery and wonder is hinted at in depictions of dyslexic-experience (Granger, 2010; Titchkosky, 2007). And, yet there is little that allows mystery and wonder to enchant the professionalization of reading. Dyslexic influence may appear ridiculous in the face of the authority of the professionalization of reading whose commitment is to understanding reading as a decoding of its component parts. For instance, the Ontario *Right to Read* report simply states that anything other than its science of reading is discreditable and will harm the new reader:

Balanced literacy or comprehensive balanced literacy approaches, cueing systems and other whole language beliefs and practices are not supported by the science of reading. They have been discredited in many studies, expert reviews and reports on teaching all students to read words … Students most at risk for reading failure, including students with reading disabilities and many students from other Code-protected groups, will not develop critical early reading skills when these approaches are used in schools. (OHRC, 2022, p. 26 *no research citations provided for claims*.)

Most nation states employ mechanisms to assemble and disseminate literacy rates together with mechanisms to identify individuals who have reading difficulties (UNESCO, n.d.). Contemporary empirical research proceeds by mapping literacy rates and by clarifying diagnostic mechanisms and treatment protocols to deal with any literacy troubles. In North America, the International Dyslexia Association (n.d.) and Dyslexia Canada (n.d.) are advocating that the category “dyslexia” be employed more regularly and be codified in educational policy to solidify the existence of reading difficulties as a medical problem in need of professional intervention. Within the framework of the professionalization of reading, and its belief in, and promotion of a science of reading, much work is being done to stipulate a wide and far reaching trouble with reading accuracy, miscues and mistakes, and thereby make the prevalence of the problem of dyslexia seem more certain. Such work not only crystallizes dyslexia as an objective identity, but it also forms the meaning of the act of reading itself, making the illusory norm or goal of constant clarity overwrite any struggle as only a reading-problem.

Struggle, mistake, and confusion remain an integral part of reading despite its professionalization. Professionalization shifts the focus from what is read and its meaning to how we read with the aim of judging accuracy and inaccuracy displayed by an individual reader. Still, having accurate knowledge of the correct place name (Iowa or Ohio), even if known with absolute certainty, would not put readers, or writers, in touch with the meaning of this symbolic communication of my place, let alone “my problem.” Why read, or pretend to read, unless there is something more to reading than accuracy and certainty? The danger of dyslexia is the danger of recognizing this “something more” in reading and this is the danger that the professionalization of reading seeks to quell. Dyslexia, even as it is a category of literate times, reveals the essential co-constitutional phenomena of the act of reading within literate culture. Reading, therefore, is a precarious act, one steeped in a human communicative relation replete with struggle, mistake, and confusion.

## Concluding Remarks

I conclude with some remarks on what my consideration of dyslexia suggests about interpretive methods.

Dyslexia, as something perceived as well as a form of perception, is an interpretive act. Part of what comprises this act, is that dyslexia is perceived as representing a disruption to the normative flow of interpretation. Exploring the category of dyslexia through interpretive methods allows us to address the way struggle, mistake and confusion, and other reading difficulties are described and experienced. This invites us to draw out norms of accuracy and values of certainty as they play out in the ordinary flow of the interpretive movement of everyday life, including the flow of reading.

Attending to the historical, professional, or personal identification of dyslexia, then, can be understood as itself a method to uncover the social processes of interpretation. Thus, the first move in interpretive methods is to treat categories of interpretation, such as dyslexia, as themselves interpretive rather than as objectively given facts. In contrast to traditional methods of inquiry where terms, such as dyslexia, are operationalized and various hypotheses are generated and pursued, interpretive methods considers the multiple ways a category has *already* been defined to explore the living social interpretive reality as it constitutes the meaning of human interaction including, in this case, the meaning of reading. At the same time, the personal experience of dyslexia is not treated as the “real truth,” whereby the Dyslexic becomes the next expert to move into the country of professionalism. Narrating subjective experiences of dyslexia is, however, a way to loosen the hold that objective renderings of it have on us. Dyslexia, like everything else that allows us to make sense of human embodiment, is forever enmeshed in endlessly layered scenes of interpretive activity (see Healey, 2021 as this relates to blindness).

Engaging such acts of sense-making, provides for the possibility of lament, for political action, and also for hesitation and theorizing. As Rod Michalko (1998) suggests in his theorizing of blindness, the least normal thing we can do is attend to normalcy’s production. In the face of reading difficulties within mass literacy, I have struggled to discern the version of normalcy produced and sustained through the sense-making device of dyslexia. Another important aspect of interpretive methods, then, is that it not only treats categories of sense-making as interpretive but also reveals *how* these categories gain authority in relation to normalcy. Instead of reasserting the primacy of normalcy, we can study how it is enforced and we can attend to the forms of enforcement (Davis, 1995). Furthermore, by conducting what might be understood as a phenomenology of the concept of dyslexia, it is possible to reveal the movement of interpretation in the making of the meaning of human life.

The final move I will highlight in this version of interpretive methods, may, ironically, be its first. It is to regard our categories of human difference as categories *of* a different way of perceiving. This move is based on the understanding that dyslexia, together with all categories of human difference, is always more and other than what society makes it and believes it to be (Stiker, 1999, p. 52; for more about this more see Titchkosky et al., 2022, pp. 1–17). It is, after all, categories of interpretation that bring us into the world as particular types of people. In this sense, we *are* our categories. Yet, we are not necessarily limited to living these categories in the ways prescribed by institutions such as those insisted on by the professionals of reading. Interpretive categories, such as dyslexia, can themselves be “read” as an occasion to recognize their limitations and, go on, to make more of dyslexia than is already prescribed for us. Being made by the words of the world that say we are not at one with its workings means to be in touch with some difference that can help us perceive the workings of this world (Freire, 1983). Dyslexia may be a difference that allows us to read the words that have created difference in the first place.

## Notes

1 *Chiasma* is not a dyslexic rendering of “chasm.” Instead, *chiasma* refers to the crosswise relation between concepts and structures that rely on each other for their sense and existence, for example, reading and readers; subject and object.

2 At the same time, there are many other competing definitions of reading and reading troubles. We can note, for example, that voluntary community-based reading programs have been regarded as a mechanism for the “pedagogy of the oppressed,” suggesting too that reading can be a form of counter-socialization (Ferri & Connor, 2006; Freire, 1983; Graff, 2017; Heap, 1991). Also, some professionals refute the empirical validity of the term (Davies, 1998; Elliott & Grigorenko, 2014), and some aim to suggest that dyslexia is a condition that can be ameliorated and, therefore, should not be understood as a developmental or permanent disability (Barden, 2011; Sudek & Encinas, 2019).

3 Prevalence of dyslexia is quantified as 5%, 10%, 15%, and even 20% of the population, or as “the most common learning disability” (OHRC, 2022, p. 9). These highly variable prevalence rates are related to conceptions of reading and reading problems, for example, “Wagner … proposed that no single prevalence estimate is correct, but rather that there is a distribution of prevalence as a function of severity.” (Wagner et al., 2020, p. 5). Of course, severity is a function of cultural conceptions of reading, poorly or otherwise. At bottom, the objectification of prevalence as it constitutes people as problems is what is prevalent!

4 The OHRC report is influencing curriculum development in every subject across the province of Ontario (Caughill, 2024). Its sense of problem readers and the problem of reading is supported by these dramatic and expansive claims which are based on a few papers (Livingston et al., 2018; Metsala et al., 2017; Partanen & Siegel, 2014; Riddick, 2010; Snowling et al., 2000.) Regarding dyslexia as a gateway to deviance further supports OHRC’s (2022, p. 7) position that “The inquiry is not just about an equal right to read—it is about an equal right to a future.” The province-wide communication of a connection between dyslexia and a presumably questionable future is our present institutionalized reality (Alexander-Passe, 2003; Davies, 1998; Dyslexia Canada, n.d.).

5 This phenomenology of the concept is related to the Deleuzoguattarian sense of the “pedagogy of the concept,” in so far as both approaches share an interest in examing how a concept appears and what it does and for whom, or what it requires of those practiced in its use (Döring, 2021; Goodley, 2007; M. A. Peters, 2004; Rajchman, 2014; Titchkosky, 2020).
